# Clinical and Laboratory Parameters of Gonadotropin-Releasing Hormone Analog Treatment Effectiveness in Children with Precocious Puberty

**DOI:** 10.6061/clinics/2019/e1205

**Published:** 2019-10-30

**Authors:** Lívia Grimaldi Abud Fujita, Heloísa Marcelina da Cunha Palhares, Adriana Paula da Silva, Janaíne Machado Tomé, Maria de Fátima Borges

**Affiliations:** Disciplina de Endocrinologia, Universidade Federal do Triangulo Mineiro (UFTM), Uberaba, MG, BR.

**Keywords:** Gonadotropin-Releasing Hormone, Puberty, Therapeutics, Luteinizing Hormone, Follicle Stimulating Hormone, Estradiol, Growth

## Abstract

**OBJECTIVE:**

There are no doubts about the clinical benefits of treatment with GnRH analogs for patients diagnosed with central precocious puberty (CPP). However, laboratory monitoring of CPP is still a matter of considerable controversy in the literature. Therefore, the main objective of this study was to evaluate the cut-off values of stimulated LH that determine gonadotrophic suppression.

**METHODS:**

Twenty-four girls, on treatment with leuprorelin acetate (LA) at 3.75 mg IM every 28 days, were studied. The clinical parameters used to indicate clinical effectiveness were regression or maintenance of sexual characteristics according to the Tanner stage, growth velocity reduction, reduction or maintenance of the difference between bone age and chronological age and maintenance or improvement of the final height prediction. For the laboratory effectiveness test, basal estradiol, LH, and FSH levels were collected before and 1 and 2 h after the administration of 3.75 mg LA.

**RESULTS:**

Eleven girls showed improvement in all clinical parameters, and their effectiveness tests were compared to those of the other patients to calculate the cut-off values, which were ≤3.64 IU/L (*p*=0.004*) for LH after 1 h and ≤6.10 IU/L (*p*<0.001*) for LH after 2 h.

**CONCLUSION:**

The LH response after the LA stimulation test, associated with clinical data and within a context of CPP, constitutes a reliable and feasible resource and can assist in monitoring the effectiveness of treatment.

## INTRODUCTION

Precocious puberty (PP) is classically defined as the development of secondary sexual characteristics before eight years of age in girls and before nine years of age in boys (1,2). Central precocious puberty (CPP) is a relatively rare condition that can interfere with somatic (3-5) and psychosocial (6-11) development in affected children. The current treatment of choice for CPP consists of the administration of depot GnRH analogs (GnRHa). These analogs’ clinical effectiveness has been demonstrated by several authors and includes final height improvement, regression of sexual characteristics according to Marshall and Tanner’s classification, decrease in growth velocity, and decrease in bone age (BA) advancement (3,4,12-17), as well as delaying the age of menarche (18-20). Thus, treated children develop puberty at a similar time as their peers.

Although the treatment clinical parameters seem highly clear, they are subjective and depend on the examiner. Therefore, many authors indicate the need for a laboratory effectiveness test that can more accurately determine gonadotropic axis suppression (12-15,20).

The classic CPP treatment monitoring test consists of collecting basal LH and FSH every 30 minutes (up to 120 minutes) after the intravenous (IV) administration of synthetic GnRH (12,21-22). In addition to being time consuming, expensive and requiring venous access, the acquisition of synthetic GnRH has become highly difficult, especially for services within the Brazilian Unified Health System (SUS).

Another piece of data that generates controversy in the literature is the cut-off value of stimulated LH, which indicates pubertal suppression. Several authors using different laboratory methods have suggested different cut-off values (12-15,20).

Therefore, the primary objectives of this study were to evaluate the clinical and laboratory effectiveness of GnRHa treatment for CPP and to determine cut-off values for the hormones measured in the GnRHa test, which determine gonadotropic suppression.

## MATERIALS AND METHODS

This study was approved by the Research Ethics Committee of Universidade Federal do Triângulo Mineiro (UFTM). Data collection was only carried out after the term of assent was read and the free and informed consent form was signed by the parent/legal guardian of the children participating in the study.

The inclusion criteria were girls with clinical aspects of CPP, whose diagnosis was confirmed by the classic GnRH stimulation test or by a GnRHa test and who would be treated with depot GnRH analogs, intramuscularly, administered every 28 days (19-23). Patients with peripheral PP were excluded, as were patients with other endocrinopathies or systemic diseases that would interfere with the results. On the basis of these criteria, 24 girls followed at the Department of Endocrinology Outpatient Clinic of UFTM were enrolled.

A clinical diagnosis of CPP was considered if the patient had at least two of the following aspects: progression of sexual characteristics according to Marshall and Tanner (23), advanced BA and growth velocity over 6 cm/year. The age of diagnosis was 6.9±1.2 years.month (yr.mo) [4.1-7.10]. Thelarche was the first sexual characteristic to appear in 23/24 girls between 3.6 and 7.5 yr.mo, with a delay in diagnosis ranging from 5 to 32 months. The patients also had growth velocity increase and BA advancement evaluated by Greulich and Pyle’s method (24).

Patients had a diagnosis of CPP confirmed by the classic acute GnRH stimulation test (n=12) by IV infusion of 100 µg of gonadorelin (Relefact^®^, Sanofi, Germany) or by a GnRHa test (n=12) via IM administration of 3.75 mg of LA (Lectrum^®^, Sandoz from Brazil, Cambé/Pr, Brazil), which is not a standard of diagnosis, after the acquisition of synthetic acute GnRH became difficult. The cut-off values for the LH peak during the tests used as indicative of activation of the gonadotrophic axis were 3.3 IU/L and ≥10 IU/L according to previous studies performed in the same service (25,26) and in the literature, respectively (12,20). After diagnosis of CPP, the patients had been treated or were undergoing treatment with an intramuscular (IM) injection of leuprorelin acetate (LA) at the initial dose of 3.75 mg every 28 days (15-20).

Regarding the etiology, all of the children had normal pituitary imaging via nuclear magnetic resonance. In 22 cases, the CPP was considered idiopathic. However, in two cases, periventricular arachnoid cysts with enlarged lateral ventricles were found, and the PP was considered organic. However, there were no pituitary hormone deficiencies of consequence.

The study participants were assessed in the beginning and during treatment at a time close to the effectiveness test performance. The clinical parameters of treatment effectiveness evaluated were regression or maintenance of the pubertal stage according to Tanner (23), decrease in growth velocity, reduction or maintenance of the difference between BA and chronological age (CA) (ΔBA-CA) and improvement of the final height prediction.

The effectiveness test was performed by measuring LH and FSH before and 1 and 2 h after LA administration at a dose of 3.75 mg IM. The variation between the lowest and the highest LH values (ΔLH) and the LH/FSH ratio at all moments of the test were also assessed. The stimulated LH cut-off value considered to be indicative of gonadal suppression, used until then in the outpatient clinic, was <6.6 IU/L, as suggested by Brito et al. (12). To determine new cut-off values for effectiveness testing, the analyses performed in girls who showed improvement of all clinical parameters were compared to the others. Moreover, the hormone levels measured at different test moments were compared to each other to assess the need to extend the test up to 2 h.

To verify whether the hormone levels measured in patients suppressed by the treatment reached prepubertal values, a control group was selected, consisting of 11 patients followed due to isolated precocious thelarche (n: 10) or isolated precocious pubarche (n: 1), who were considered “variants of normal” but shared the same phenotype and in whom a laboratory diagnosis of CPP was ruled out. Each variable studied in the effectiveness test of the girls undergoing treatment (and suppressed) was compared with the variables measured in the stimulation test of the control group patients (performed to rule out CPP).

LH and FSH were measured using Elecsys Assay commercial kits (Roche Diagnostics GmbH, Mannheim, Germany) according to the electrochemiluminescence method (ECLIA) and analyzed in a Cobas 6000 and 601 automated system (Roche Diagnostics, Mannheim, Germany). The minimum detection value of the method is 0.1 IU/L for both hormones. LH has inter- and intra-assay variabilities of approximately 2% each. FSH has an interassay variability of up to 4.5% and intra-assay variability of up to 2.8%. Estradiol was also measured using the Elecsys Estradiol III Assay (Roche Diagnostics GmbH, Mannheim, Germany) through ECLIA and analyzed by the same automated system as the above mentioned hormones. The method’s lower limit value is 18.36 pmol/L, and its intra- and interassay variability values are up to 6.7% and 10.6%, respectively.

### Statistical Analysis

The data are presented as the mean ± SD and range. Normal distribution was analyzed using the Shapiro-Wilk test, and for comparisons of two independent samples (such as basal and stimulated LH and FSH during treatment *vs* the same parameters of the normal control group), the Mann-Whitney test was used. To determine the levels of gonadotropic suppression, a receiver operating characteristic (ROC) curve analysis was performed for each test variable when testing the treatment effectiveness. Anthropometric clinical data obtained after six months of treatment with GnRHa were considered the “gold standard”, and controlled vs uncontrolled patients were evaluated. The sample size for the ROC curve was obtained according to Arango (27) and calculated with the aid of DIMAM 1.0 software. (Guanabara Koogan, Rio de Janeiro, RJ, Brazil, version 2005).

To establish whether it was actually necessary to perform the hormone measurements 2 h after the GnRHa testing, the hormone levels measured at the different moments were compared using Friedman’s test. Comparisons and data from the ROC curve analysis were obtained using SPSS version 20 and Medcalc 10.3 software, respectively. The observed differences were considered significant when the significance level (*p*) was less than 0.05.

## RESULTS

At the start of treatment, the girls’ mean CA was 7.8±1.4 years.month (yrs.mo). The mean BA was 9.11±1 yrs.mo (4.5-9.5). The ΔBA-CA at the start of treatment was, on average, 25.2±15.1 (-19-53). The mean growth velocity (GV) at the start of treatment was 9.0±3.24 cm/year (3.0 to 18.0 cm/year). Auxological parameters at diagnosis and during treatment are represented in Table 1.

All of the patients were treated with a standard dose of 3.75 mg depot LA (Lectrum^®^, Sandoz) IM every 28 d; the initial dose per weight was 130.0±49.7 μg/kg and ranged from 70.5 to 292.9 μg/kg. To determine whether LA treatment was effective, the gonadotropin-stimulation test was performed. At that time, the patients’ clinical characteristics were studied. The mean time between the start of treatment and the effectiveness test performance was 10.3±4.8 months. The mean CA was 8.9±1 yr.mo, whereas the mean BA was 11.1±1.4 yr.mo. The mean ΔBA-CA was 26.5±17.6 yr.mo (Table 1).

At the reassessment, the mean LA dose according to the patients’ weights decreased to 111.8±38.7 μg/kg (from 66.7 to 234.4 μg/kg). Individualized clinical and laboratory data aimed to determine treatment effectiveness are presented in Table 2. To calculate GV prior to reassessment, the last 6-month period of treatment was considered in most of the patients, and it was found that the mean GV decreased to 6.0±2.3 cm/year and the GV standard deviation to 0.6±2.6. Eight patients (30%) did not attain initial assumed adequate laboratory suppressed criteria (<6.6 IU/L) (Table 2), which could be due either to lack of adherence or the need for a large dose of LA. After ensuring that the children were receiving their medication regularly, it was adjusted by reducing the interval between doses to 21 d, considering that this option was the one available in Brazilian Public Services.

Another question raised by the present study was whether it would be necessary to extend the test with GnRHa up to 2 h. Hormone measurements were compared to each other at different test moments, and it was found that basal LH values showed significant differences between LH values after 1 h and after 2 h of testing (*p*<0.001*). There was no significant difference between LH values after 1 h and LH after 2 h of testing (*p*=0.164). Conversely, the values of the FSH and LH/FSH ratio found in the effectiveness test differed from each other (*p*<0.001* between FSH times and *p*<0.001* for LH/FSH ratio times).

**Figure 1:**
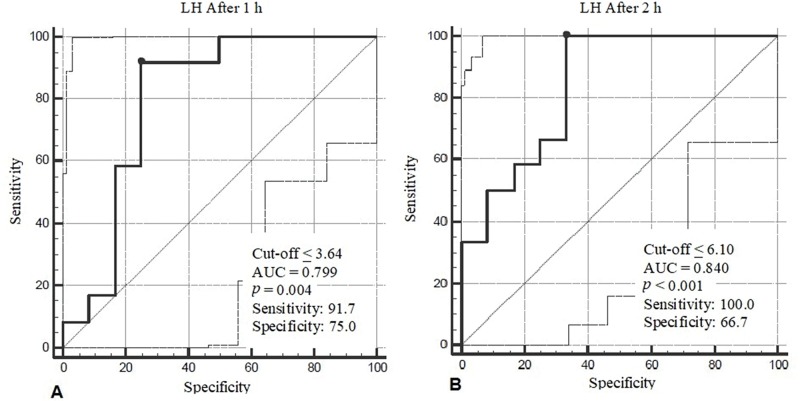
ROC curves of LH levels measured at 1 h (A) and 2 h (B) after intramuscular administration of 3.75 mg LA (GnRH analog test). AUC: Area Under the Curve.

Basal estradiol values in patients with pubertal suppression were ≤18.36 pmol/L with a sensitivity (S) of 91.7% and specificity (Sp) of 50.0% (*p*=0.032*). LH values after 1 h of testing showed a cut-off value of ≤3.64 IU/L (S: 91.7% and Sp: 75.0%; *p*=0.038*), while LH after 2 h of testing showed a cut-off value ≤6.10 IU/L (S: 100% and Sp: 66.7%; *p*<0.001*), which is represented in Figure 1.

The ΔLH showed a cut-off value ≤6 IU/L (S: 100% and Sp: 58.3%; *p*<0.001*). FSH also showed statistical significance for its stimulated levels, and its cut-off value after 1 h of testing was ≤5.43 IU/L (S: 83.8% and Sp: 75.0%; *p*=0.024*), and after 2 h of testing, this value was ≤8.31 IU/L (S: 83.3% and Sp: 75.0%; *p*=0.042*). The other variables studied in the test were not statistically significant. Table 3 shows the cut-off values for the effectiveness test.

Finally, to evaluate whether the patients with a CPP diagnosis who showed improvement in all assessed clinical parameters had hormone values after the stimulation test with GnRHa that were as low as those in the control group, each test variable of these two groups was compared between them. Except for basal estradiol and basal FSH, all other variables showed significant differences between the groups. Table 4 shows these results.

## DISCUSSION

There are no doubts regarding the need for CPP treatment with GnRHa and its clinical effectiveness (1-20). However, the laboratory monitoring of CPP treatment still generates controversy in the literature, specifically with regard to LH concentrations indicating the cut-off value that would define suppression of the hypothalamus/pituitary axis, which in association with an expected behavior of clinical parameters would convey the effectiveness of the treatment. In our study involving 24 girls with CPP and employing ECLIA as a method to assay hormonal concentrations, we identified values of LH ≤3.64 UI/L at the first hour and ≤6.10 UI/L at the second hour of the depot LA stimulation test as cut-off values that would reliably indicate suppression of the hypothalamus/pituitary axis. Unfortunately, we could not compare these figures with the “gold standard” test using synthetic GnRH, which is used to determine gonadotropic axis activation. Conversely, we had the opportunity to compare our data with those from a natural control group (26) of 11 girls with isolated premature thelarche, who, in general, shared clinical and laboratory similarities with prepubertal children.

Despite, as previously mentioned, the subjectivity of the clinical parameters related to puberty treatment follow-up, it was necessary to use the clinical examination as the basis for calculating gonadal hormone suppression cut-off values, since synthetic GnRH employed to test the LH response and monitor treatment became unavailable. To increase the accuracy of this examination, each patient was assessed at each consultation by three experienced pediatric endocrinologists who always reached a consensus on the Tanner stage. They also jointly assessed the patients’ BA by X-ray. At each consultation, the height of the patients was measured three times, and the average of the three measurements was used. In addition to careful clinical examination, clinical improvement with treatment was considered only when all clinical parameters (Tanner stage, GV, difference between BA and CA, and final height prediction) improved or did not worsen.

Several different cut-off levels of LH after the LA test have been reported in the literature and have shown great variations related to the LH assays and the time of sampling (12,21,28,29). In fact, there is no clear cut-off for the diagnosis of CPP or for the monitoring of GnRH treatment (30).

Brito et al. (12), on the basis of 16 clinically well-suppressed girls with CPP by a standard 3.75 mg of LA, defined the cut-off LH concentration as <6.6 IU/L 2 h after GnRHa injection. Despite using the fluoroimmunometric assey (FIA) method, this cut-off is closest to ours at 2 h. Dermibilek et al. (30) in a larger sample of 142 girls well-controlled by a standard depot LA treatment comparable to ours and using FIA, the method closest to ECLIA, reported a cut-off of 2.5 IU/L. However, these researchers chose to sample a single LH measurement at 90 minutes. The researchers reported concordance between these values and those derived from the classic acute GnRH stimulation test, although the LH peak in this test occurs in general at 60 minutes (25). Perhaps our differences in suppression were due to the time chosen for sampling because our numbers at 1 h were close to those found at 90 minutes, and the ROC curves reported by Dermibilek et al. (29) showed the best sensitivity and specificity probably related to the largest number of patients. Our LH values of ≤3.64 IU/L after 1 h of testing and ≤6.10 IU/L after 2 h of testing showed good sensitivity and specificity in addition to statistical significance. These values are close to those suggested in the literature, corresponding to 4.0 IU/L after 40 minutes of testing (13) and 6.6 IU/L after 2 h of testing (12). ΔLH values were not higher than 6.00 IU/L in suppressed patients, but ΔLH should be used with caution as a parameter of treatment monitoring because it is not reproducible.

In relation to the time of sampling, Bhatia et al. (21) demonstrated that LA, despite being a depot and extended-release medication, has free particles in its preparation and can stimulate gonadotropin release 7.5 minutes after its administration. The LH peak is sustained between 30 and 120 minutes, and a single measurement during that interval is sufficient to represent its maximum level after leuprorelin administration. Conversely, FSH levels show a progressive increase up to 120 minutes after administration (21). Following this line of reasoning, many experts have suggested that the effectiveness test of CPP treatment be performed at 2 different moments before and 60 minutes after the administration of leuprorelin (13-15,20).

The results of the present study showed that there is no significant difference between the levels of LH collected 1 h after the test and those collected after 2 h. Conversely, FSH (and LH/FSH ratio) showed a significant difference at all test moments, showing that FSH levels actually remain on the rise for 2 h after leuprorelin administration, as demonstrated by Bhatia et al. (21).

The pattern of FSH response during the acute GnRH and the LH/FSH ratio of approximately 1 IU/L has been used by some authors for CPP diagnosis, and in cases of gonadotropin-independent sexual precocity, FSH does not increase significantly during the test, whereas its elevation is more significant in CPP (31). To determine treatment effectiveness, FSH measurement is also routinely performed during the test, but there are no references for values that determine gonadotrophic axis suppression. FSH levels ≤5.43 IU/L after 1 h of testing were indicative of pubertal suppression with statistical significance, as were FSH levels ≤8.31 IU/L after 2 h of testing. Girls with good clinical control tended to have a basal LH/FSH ratio ≤0.27; after 1 h, ≤2.02; and after 2 h, ≤0.89.

To finalize the analysis of the variables measured during the effectiveness test with LA, we assessed whether the values obtained in patients with effective pubertal suppression were equal to prepubertal values. To answer this question, the tests performed in patients with clinical effectiveness were compared with the tests of control patients, namely, those with isolated precocious thelarche. Each variable measured by the test was compared individually.

Except for basal estradiol and basal FSH, which showed no difference between treatment-suppressed and control patients, all other variables (basal LH, stimulated LH, ΔLH, stimulated FSH and all moments of LH/FSH ratio) were statistically higher in patients treated and suppressed with LA. Thus, effective treatment did not recover the prepubertal gonadotropin levels. Consequently, the cut-off values of the variables obtained at LA testing differ in determining the diagnosis of CPP and laboratory effectiveness of the treatment.

This study has a main limitation, that is, the small sample size that reduced the accuracy and reliability of cut-offs recommended for monitoring treatment, and consequently, studies with larger samples may yield different results. On the other hand, its strength is to show that considering that the test is performed in children, being fast and practical are important qualities, and the collection of basal LH values and LH values after 1 h of testing seems to be the best option. In this case, values higher than 3.64 IU/L indicate treatment adjustment. However, if it is decided to collect LH after 2 h of testing, the cut-off value used is a different one (6.10 IU/L) and similar to the findings of Brito et al. (12).

In conclusion, the LH response after an LA stimulation test, associated with clinical data and within a context of CPP, constitutes a reliable and feasible resource and can assist in monitoring the effectiveness of treatment.

It is also possible to conclude that FSH measurement aimed at evaluating treatment effectiveness is not necessary. Finally, effective treatment with GnRHa does not reach the prepubertal levels of stimulated LH.

## AUTHOR CONTRIBUTIONS

Fujita LGA, Palhares HMC and Borges MF developed the project, coordinated the work and wrote the manuscript, as well as, were responsible for careful treatment and follow-up of the patients. Tomé JM and Silva AP collected the patient records, plotted the data in the Excel tables, performed the statistical analysis and configured the tables and references.

## Figures and Tables

**Table 1:** Clinical and laboratory data from children with PP.

Clinical data	At the diagnosis	During treatment	Control group
N	24	24	11
CA (years)	6.9±1.2 (4.1-7.10)§	8.9±1.3 (5.8-10.4)	6.2±1.2 (3.2-7.4)
Height Z-score	0.85±1.25 (-2.04-3.13)	1.04±1.15 (-1.45-3.25)	0.89±0.85 (-0.68-2.16)
BMI Z-score	0.72±1.36 (-2.55-2.89)	0.91±1.39 (-1.97-3.31)	1.46±1.67 (-1.10-4.69)
BA (years)	9.0±1.8 (5.9-11.0)	11.1±1.4 (8.0-13.0)	7.4±2.3 (3.6-11.0)
ΔBA-CA (years)	2.0±1.3 (-1.7-3.8)	2.2±1.5 (-1-7.3)	1.4±1.3 (0.2-3.8)
GV (cm/year)	9.3±2.3 (3.0-18.0)	6.0±2.3 (1.8-11.0)	7.4±2.3 (5.8-11.16)
GV-SD	4.0±3.3 (0.3-14.1)	0.6±2.6 (-4.4-6.2)	1.8±2.6 (-0.4-6.0)
Basal estradiol (pmol/L)	20.41±13.14 (4.00-63.14)	30.18±37.26 (18.36-196.03)^a^	22.14±9.07 (18.36-46.99)^a^
Basal LH (UI/L)#	4.86±3.10 (0.23-9.67)	1.28±2.05 (0.10-7.83)^b^	0.12±0.11 (0.01-0.44)^b^
LH after 1 h (UI/L)#	32.05±21.38 (3.81-67.79)	6.96±9.83 (1.50-44.37)^c^	1.66±0.80 (0.35-2.66)^c^
LH after 2 h (UI/L)#	27.17±14.75 (6.84-49.88)	10.09±18.17 (0.90-80.14)^d^	2.04±0.87 (0.40-3.34)^d^
Δ LH#	28.25±19.15 (6.61-64.21)	9.04±16.45 (1.13-74.18)^e^	1.86±0.99 (0.25-3.24)^e^
Basal FSH (UI/L)#	6.41±2.23 (2.89-9.93)	2.24±1.88 (0.32-7.41)^f^	1.82±1.05 (0.18-3.45)^f^
FSH after 1 h (UI/L)#	14.54±5.44 (6.13-25.58)	5.57±3.76 (1.13-16.76)^g^	8.98±4.85 (1.76-19.88)^g^
FSH after 2 h (UI/L)#	15.81±13.86 (10.25-24.97)	8.46±6.12 (1.24-25.15)^h^	14.04±5.77 (2.38-25.48)^h^
Basal LH/FSH#	0.73±0.35 (0.24-1.32)	0.49±0.56 (0.04-2.22)^i^	0.12±0.15 (0.01-0.55)^i^
LH/FSH after 1 h#	2.00±0.95 (0.62-3.50)	1.34±1.12 (0.41-4.32)^j^	0.22±0.13 (0.05-0.54)^j^
LH/FSH after 2 h#	1.65±0.61 (0.67-2.82)	1.06±0.96 (0.36-3.52)^k^	0.18±0.11 (0.03-0.43)^k^

N: number of patients; CA: chronological age; BMI: body mass index; BA: bone age; ΔBA-CA: difference between bone age and chronological age; GV: growth velocity; GV-SD: growth velocity - standard deviation;

# during the LA test; Δ LH: difference between the highest and the lowest measured LH value;

§ numbers expressed as the mean ± SD (minimum - maximum values).

Mann-Whitney test: letters overwritten in data pairs indicate that they were compared; only h: *p*>0.05.

**Table 2:** Cut-off values of the studied variables in the effectiveness test with LA indicating laboratory suppression.

Variable	Cut-off value	Sensit. (%)	95% CI	Specif. (%)	95% CI	AUC	*p*
Basal E2#	≤5.00	91.7	61.5-99.8	50.0	21.1-78.9	0.694	0.0318*
Basal LH§	≤0.35	75.0	42.8-94.5	66.7	34.9-90.1	0.625	0.3292
LH after 1 h	≤3.64	91.7	61.5-99.8	75.0	42.8-94.5	0.799	0.0038*
LH after 2 h	≤6.10	100	73.5-100.0	66.7	34.9-90.1	0.840	<0.0001*
Δ LH	≤6.00	100	73.5-100.0	58.3	27.7-84.8	0.854	<0.001*
Basal FSH§	≤2.94	100	73.5-100.0	41.7	15.2-72.3	0.667	0.1772
FSH after 1 h	≤5.43	83.8	51.6-97.9	75.0	42.8-94.5	0.750	0.0236*
FSH after 2 h	≤8.31	83.3	51.6-97.9	75.0	42.8-94.5	0.785	0.0042*
Basal LH/FSH	≤0.27	83.3	51.6-97.9	58.3	27.7-84.8	0.618	0.3635
LH/FSH after 1 h	≤2.02	91.7	61.5-99.8	33.3	9.9-65.1	0.573	0.5523
LH/FSH after 2 h	≤0.89	83.3	51.6-97.9	50.0	21.1-78.9	0.632	0.2681

# E2: expressed in pmol/L;

§ LH and FSH: expressed in UI/L; Sensit.: sensitivity; Specif.: specificity; AUC: area under the curve; h: hour; 95% IC: 95% confidence interval;

* *p*<0.05.

**Table 3:** Evaluation of the effectiveness of PP treatment with a GnRH analog (3.75 mg, IM, every 28 days) according to clinical and laboratory data of interest.

Case n°	Tanner (B)		GV (cm/month)		ΔBA-CA (months)		FHP (cm)		LH (IU/L)#
	BT	DT	BT	DT	BT	DT	BT	DT	
1	2	2	0.58	0.50	27	38	151.9	148.8	4.6
2	3	3	0.60	0.46	34	34	148.9	150.0	4.64
3	3	3	0.70	0.60	37	25	143.9	152.0	5.54
4	2	2	1.30	0.38	35	39	148.7	149.8	9.65
5	3	3	0.56	0.48	42	32	156.6	163.2	6.1
6	2	1	0.25	0.66	22	25	133.1	136.4	6.74
7	3	3	0.95	0.80	-19	-12	157.5	158.3	6.96
8	4	2	0.86	0.40	47	41	152.1	155.0	5.16
9	2	2	0.82	0.44	27	40	153.6	150.8	3.58
10	3	3	0.90	0.92	14	22	163.4	164.2	54.48
11	3	1	0.77	0.63	32	21	146.5	152.6	2.83
12	2	1	0.50	0.12	31	27	150.4	151.0	4.43
13	2	1	0.60	0.50	14	19	159.3	157.5	9.98
14	4	3	0.66	0.36	30	29	160.1	161.1	80.14
15	3	3	0.63	0.20	36	20	152.5	156.4	2.66
16	3	3	0.53	0.28	28	25	154.5	158.0	3.8
17	1	2	0.80	0.77	1	28	165.1	150.5	4.11
18	3	3	0.50	0.50	23	15	152.5	158.3	3.44
19	3	1	0.85	0.15	19	8	155.0	158.2	3.32
20	2	1	1.10	0.38	53	87	149.8	133.1	7.97
21	3	1	0.75	0.57	13	30	166.2	157.2	2.97
22	2	3	1.50	0.60	33	30	151.2	157.3	7.0
23	3	1	0.76	0.40	17	19	144.0	140.9	0.9
24	2	1	0.60	0.36	8	4	163.2	164.7	1.24
Mean	-	-	0.75	0.48	25.2	26.9	153.3	153.6	10.09
SD	-	-	0.27	0.20	15.4	17.6	7.6	8.0	18.2
Minimum	-	-	0.25	0.12	-19	-12	133.1	133.1	0.9
Maximum	-	-	1.50	0.92	53	87	166.2	164.7	80.14

B: breast; BT: beginning of treatment; DT: during treatment; GV: growth velocity; ΔBA-CA: difference between bone age and chronological age; FHP: final height prediction;

# LH 2 h after GnRH analog treatment.

**Table 4:** Comparisons between the variables of the stimulation test with LA, 3.75 mg IM, of the patients with CPP who showed improvement of all assessed clinical parameters and the patients in the control group.

Variable	Median (P25; P75)		
	Control	Suppressed patients	*p*
Basal E2 (pmol/L)	18.36 (18.36; 18.65)	18.36 (18.36; 18.36)	0.633
Basal LH (IU/L)	0.10 (0.10; 0.10)	0.33 (0.17; 0.81)	0.005*
LH after 1 h (IU/L)	1.75 (1.03; 2.23)	3.09 (2.87; 3.45)	0.001*
LH after 2 h (IU/L)	2.10 (1.57; 2.76)	3.51 (2.74; 4.60)	0.004*
ΔLH (IU/L)	2.00 (1.13; 2.69)	3.23 (2.46; 3.64)	0.007*
Basal FSH (IU/L)	2.33 (0.82; 2.55)	1.49 (1.04; 1.94)	0.588
FSH after 1 h (IU/L)	9.80 (6.84; 10.61)	3.51 (2.99; 5.07)	0.006*
FSH after 2 h (IU/L)	15.30 (11.17; 15.72)	4.57 (2.90; 7.11)	0.002*
Basal LH/FSH	0.04 (0.04; 0.15)	0.24 (0.13; 0.44)	0.001*
LH/FSH after 1 h	0.21 (0.11; 0.26)	0.96 (0.55; 1.60)	0.001*
LH/FSH after 2 h	0.19 (0.10; 0.21)	0.70 (0.46; 1.13)	0.001*

Mann-Whitney test. P25: 25^th^ percentile; P75: 75^th^ percentile; h: hour;

* *p*<0.05.
